# Detection of High- and Low-Risk HPV DNA in Archived Breast Carcinoma Tissues from Ethiopian Women

**DOI:** 10.1155/2021/2140151

**Published:** 2021-10-11

**Authors:** Endale Gebregzabher, Daniel Seifu, Wondemagegnhu Tigneh, Yonas Bokretsion, Abebe Bekele, Markos Abebe, Gabriella Lillsunde-Larsson, Christina Karlsson, Mats G. Karlsson

**Affiliations:** ^1^Department of Biochemistry, St. Paul's Hospital Millennium Medical College, Ethiopia; ^2^Department of Biochemistry, Division of Basic Sciences, University of Global Health Equity, Kigali, Rwanda; ^3^Department of Oncology, School of Medicine, Addis Ababa University, Ethiopia; ^4^Department of Pathology, School of Medicine, Addis Ababa University, Ethiopia; ^5^Deputy Vice Chancellor, University of Global Health Equity, Kigali, Rwanda; ^6^Armauer Hansen Research Institute (AHRI), Addis Ababa, Ethiopia; ^7^School of Health Sciences, Orebro University, Sweden; ^8^Department of Laboratory Medicine, Faculty of Medicine and Health, Orebro University, Sweden

## Abstract

**Background:**

Human papilloma virus (HPV) is involved in the development of cancer of the cervix, mouth and throat, anus, penis, vulva, or vagina, but it has not been much considered as a cause of breast cancer. Recently, a number of investigations have linked breast cancer to viral infections. High-risk HPV types, predominantly HPV types 16, 18, 31, 33, 35, 39, 45, 51, 52, 56, 58, and 59, are established as carcinogens in humans. In this study we aimed to detect 19 high-risk and 9 low-risk HPVs from archived breast tumor tissue among Ethiopian women.

**Methods:**

In this study, 75 breast cancer patients from Tikur Anbassa Specialized Hospital in Addis Ababa (Ethiopia) were included. HPV detection and genotyping were done using the novel Anyplex™ II HPV28 Detection Assay at the Orebro University Hospital, Sweden. The Anyplex™ II PCR System detects 19 high-risk HPV types (16, 18, 26, 31, 33, 35, 39, 45, 51, 52, 53, 56, 58, 59, 66, 68, 69, 73, and 82) and 9 low-risk HPV types (6, 11, 40, 42, 43, 44, 54, 61, and 70). IHC for p16 was done using an automated system, the Dako Autostainer Link.

**Results:**

Out of the 75 valid tests, two were found to be positive (2.7%) for HPV. One of the cases was positive for the high-risk HPV16 genotype while the other was positive both for the high-risk HPV39 and the low-risk HPV6. The cell cycle protein p16 was highly expressed in the case positive for the high-risk HPV16, but it was not expressed in the case positive for HPV39.

**Conclusion:**

The prevalence of HPV is low in Ethiopian breast cancer patients, but the role played by HPV in breast carcinogenesis among Ethiopian breast cancer patients cannot be commented based on these observations.

## 1. Background

Human papilloma virus (HPV) has been implicated in the development of cancer of the cervix, mouth and throat, anus, penis, vulva, or vagina, but it has not been much considered as a cause of breast cancer. However, a growing number of investigations have linked breast cancer to viral infections, including human papilloma virus (HPV), Epstein–Barr virus (EBV), mouse mammary tumor virus (MMTV), and human cytomegalovirus (HCMV) [1]. Human papilloma viruses (HPVs) are nonenveloped DNA viruses belonging to the Papillomaviridae family [2, 3]. Over 170 types of HPVs have been identified [3], the majority of which affect the genital tract epithelia, the mucosa of the upper respiratory tract, and the skin [2, 3]. HPVs are categorized as high risk or low risk, depending on their carcinogenic potential. High-risk HPV types cause cancer; however, low-risk types are not carcinogenic but cause benign anogenital warts and recurrent respiratory papillomatosis [3, 4]. High-risk HPV types, predominantly HPV types 16, 18, 31, 33, 35, 39, 45, 51, 52, 56, 58, and 59, are established as carcinogens in humans, while HPV68 is probably carcinogenic [5]. HPV types 16 and 18 are the most common high-risk types and are responsible for >70% of all cervical cancer cases [5]. HPVs are characterized by the presence of three functional code regions in their genome: the E region that codes the early viral function, the L region which is responsible for the late viral function, and the long control region (LCR) [2]. Even though HPVs are known to be responsible for the development of cervical cancers, HPV infections are often asymptomatic, and most sexually active individuals become infected with HPV at least once in their lifetime [3]. A recent systematic review and metanalysis by Ren et al. examined 37 case-control studies containing 3,607 breast cancer cases and 1,728 controls in a wide range of countries that compare the prevalence of high-risk HPVs in breast cancer as compared to benign breast or normal breast [6]. In their study, Ren et al. showed an increase in breast cancer risk if positive with human papillomavirus (HPV) (summary odds ratio (SOR) = 6.22, 95% confidence interval 4.25 to 9.12; *P* = 0.0002). Ren et al. also showed three high-risk HPV types (HPV types 16, 18, and 33) are positively correlated to breast cancer [6]. Similarly, a metanalysis conducted by Lawson et al. in 2015 showed that the prevalence of HPV is fourfold higher in breast cancer (21.5%) than controls (5.1%) [7].

In another study, it has been shown that women with HPV-associated cervical pathology are at increased risk from the same HPV type positive breast cancer which implies a possible link between HPV and breast carcinogenesis [8]. There is only one published study so far done to assess the prevalence of HPV in the African continent. This study from Rwanda [9] was conducted in 47 archived formalin-fixed paraffin-embedded tissues to detect and genotype HPV DNA. They reported the prevalence of HPV at 46.81% of cases. The most common genotype in this study were HPV16 (77%), followed by HPV33 (14%) and HPV31 (9%) [9].

In Ethiopia, according to one study, more than 6,000 new cervical cancer cases are diagnosed annually and HPV is the cause of most of the cases [10]. A study done in 2014 found that the most common genotype among cervical cancer patients in Ethiopia was HPV16, followed by HPV52, HPV56, and HPV31 [11]. Another study done in 2013 also found HPV16 as the most common genotype, followed by HPV52, HPV58, and HPV18 [12]. So far, there is no published study done to assess the role of HPV among breast cancer cases in Ethiopia. Therefore, in this study we aimed to detect 19 high-risk and 9 low-risk HPVs from archived breast tumor tissue to look at the prevalence among Ethiopian women for the first time.

## 2. Materials and Methods

### 2.1. Patient and Sample Characteristics

The participants involved in this study were previously investigated in our published work to characterize the molecular classification and determine distribution of the androgen receptor in breast cancer among Ethiopian women [13, 14]. The patient and sample characteristics are summarized as follows. The study initially recruited 114 cases with a pathology confirmed invasive breast carcinoma who visited Tikur Anbessa Specialized Hospital (TASH) in Addis Ababa (Ethiopia) for treatment. After obtaining a written informed consent, participant's age, tumor grade, stage of disease, and type of pathology were collected from their medical records. Formalin-fixed paraffin-embedded (FFPE) tissue samples were obtained from TASH and St Paul's Hospital Millennium Medical College (SPHMMC) pathology laboratories because some of the biopsies were tested at this hospital. All FFPE blocks were sectioned and H&E stained and evaluated at the Orebro University Hospital, Sweden. TMA were constructed for immunohistochemistry evaluation, and tumor cores for molecular testing were taken from the primary donor blocks and transferred to sterile Eppendorf tubes. A tissue core sample for PCR TMA Grand Master automated system (3DHISTECH Ltd., Budapest, Hungary) was used to sample cores for PCR analysis. A pathologist marked representative parts of the individual invasive tumor with percentage of tumor cells for sampling of tissue for PCR. 0.6-millimeter punch biopsies corresponding to the marked area were taken from paraffin blocks. For each patient, two biopsy cores were taken from the same tumor.

### 2.2. DNA Extraction

DNA was isolated from tissue core samples of each tumor specimen. The presence of malignant cells was assessed in all samples by evaluation of slides stained with hematoxylin and eosin (H&E). Genomic DNA (gDNA) was isolated from the tumor cores using the QIAamp DNA Mini Kit (Qiagen), including proteinase K treatment (Qiagen) followed by purification using the QIAcube automated system (Qiagen). The DNA concentration was measured using a Nanodrop ND-1000 (Nanodrop Technologies). The gDNA samples were stored at 4°C.

### 2.3. Multiplex Real-Time PCR

The multiplex quantitative real-time PCR was done at the Orebro University Hospital. Real-time PCR amplification was performed using the Anyplex™ II HPV28 Detection Assay (Seegene, Seoul, Korea), in accordance with the manufacturer's protocol, in a CFX96 Real-Time Thermocycler (Bio-Rad, Hercules, CA, USA). The Anyplex™ II HPV28 Detection Assay is a novel multiplex real-time PCR assay that permits the simultaneous amplification, detection, and differentiation of target nucleic acids of 28 HPV types and internal control (IC). The Anyplex™ II PCR System detects 19 high-risk HPV types (16, 18, 26, 31, 33, 35, 39, 45, 51, 52, 53, 56, 58, 59, 66, 68, 69, 73, and 82) and 9 low-risk HPV types (6, 11, 40, 42, 43, 44, 54, 61, and 70).

### 2.4. Immunohistochemistry for p16

Immunohistochemistry (IHC) for p16 was done using an automated system, the Dako Autostainer Link. Formalin-fixed paraffin sections were cut at 4 microns and rehydrated with water. Heat-induced epitope retrieval was performed with the FLEX TRS High-pH Retrieval Buffer for 20 minutes. After peroxidase blocking, the specific monoclonal antibody (source and dilution: p16 clone G175-405 from BD, USA and diluted 1/25) was applied at room temperature for 20 minutes. The FLEX + Rabbit EnVision System was used for detection. DAB chromogen was then applied for 10 minutes. Slides were counterstained with Mayers hematoxylin for 5 seconds and then dehydrated and coverslipped. Slides were scanned on a Pannoramic 250 digital scanner (3D HISTECH Ltd., Budapest, Hungary) and images scored using the software program “CaseViewer” (3D HISTECH Ltd., Budapest, Hungary). Negative controls were included in the run.

### 2.5. Statistical Analysis

Statistical analysis was done using SPSS for windows version 21. Continuous data are reported as mean ± SD or number (proportions). Skew distributions are reported as the median value with minimum and maximum.

## 3. Results

### 3.1. Patients

There were 114 participants with FFPE available for this study. Only 75 cases had valid results in the genotyping experiment for HPV. Mean age at diagnosis of the 75 cases was 42 years (SD12), and median age was 40 (range 22–75). Most of the participants (40%) were <40 years old. About 31% of the participants were ≥50 years and 28% were 40-49 years old.

### 3.2. Histopathological Characteristics

Of the 75 tumors, 7% were grade 1, 29% were grade 2, and 33% were grade 3. The stages were as follows: Stage 1—20%; Stage 2—31%; Stage 3—35%, and Stage 4—1%. The most common type of histology which is presented in Table 1 was infiltrating ductal (60%), and the lobular type was only 5% (Table 1).

### 3.3. HPV Detection

Of the 114 tumors, only 75 had detectable internal control; therefore, the rest were rejected. Out of the 75 valid tests, 2 were found to be positive (2.7%). One of the cases was positive for the high-risk HPV16 genotype, while the other was positive both for high-risk HPV39 and low-risk HPV6.

### 3.4. p16 Immunostaining

**Figure 1:**
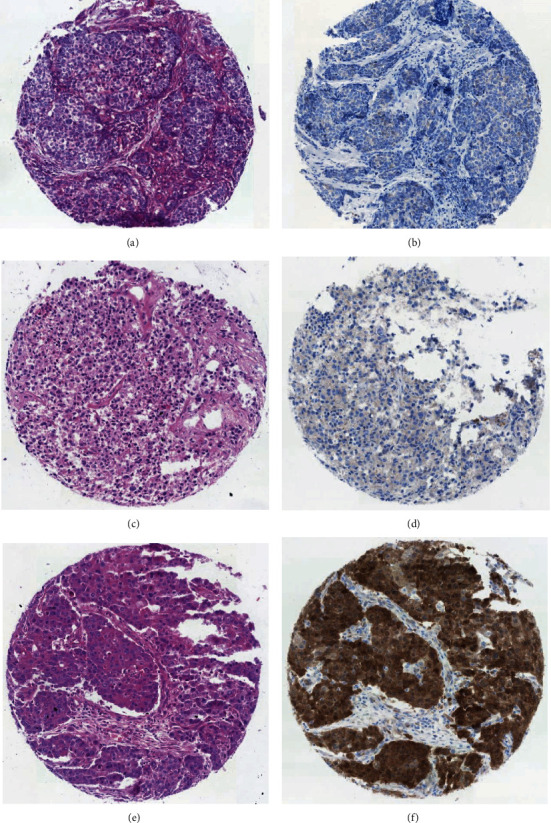
p16 immunostaining of HPV+ breast tumors with a representative HPV-negative/p16-negative breast tumor tissue. (a) H&E staining of the representative HPV-negative case. (b) Negative p16 immunostaining of the representative HPV-negative case. (c) H&E staining of the HPV39 and HPV6 dual-positive case. (d) Negative p16 immunostaining of the HPV39 and HPV6 dual-positive case. (e) H&E staining of the HPV16-positive case. (f) Strong, diffuse, nuclear, and cytoplasmic p16 immunostaining of the HPV16-positive case.

A strong expression of p16 was detected in the case positive for HPV16 but not detected in the case that was dual positive for HPV39 and HPV6. The expression of p16 across our cohorts is summarized in Table 2. Immunohistochemical staining of p16 for the two HPV-positive cases is presented in Figure 1.

## 4. Discussion

In this study, we explored the presence of 19 high-risk HPV types (16, 18, 26, 31, 33, 35, 39, 45, 51, 52, 53,56, 58, 59, 66, 68, 69, 73, and 82) and 9 low-risk HPV types (6, 11, 40, 42, 43, 44, 54, 61, and 70). The Anyplex Multiplex qPCR technology for detection and genotyping of HPVs were utilized. We detected the high-risk HPV DNA 2/75 (2.7%) of the study participants from Ethiopian breast cancer patients. Researchers from different countries reported an overall prevalence of 2–74% of HPV in breast cancer as presented in the paper by Lawson et al. [15]. Our observation is consistent with two different studies from China by Peng et al. (2%) and Li et al. (2%) [16, 17]. The variation in prevalence of HPV in breast cancer in different countries may relate to the existence of geographic differences in HPV infection [15, 18]. Lawson et al. showed that countries with low rates of HPV-associated cervical cancer appear to have high rates of breast cancer and vice versa. Hence, they argue that HPV may not play a major role in breast carcinogenesis; however, they suggested the possibility that HPVs may be involved in some but not all breast cancers [15]. Similarly, Lawson et al. refer to the work done by Grulich et al. [19] which showed that the prevalence of breast cancer is not increased in immunocompromised patients (patients with HIV infections or organ transplant recipients) as supportive evidence for an indirect and minor role of HPV in breast cancer [15, 19]. The prevalence of HPV in our study (2.7%) is significantly different than the Rwandan study (47%), and this may be due to true epidemiological difference in the distribution of HPV in the two countries or it may be attributed to a variation in methodologies applied to each of the studies [9].

Various types of HPV have been identified from different countries with HPV16 reported as the most common HPV followed by the HPV18 and HPV33 [6, 15]. In our study, the HPV type detected in breast cancer samples positive for HPV DNA was high-risk HPV16 in 1 case (1.2%) and high-risk HPV39 in another case (1.2%). One of the subjects positive for high-risk HPV39 was also positive for low-risk HPV type 6, though the low-risk HPV6 has not been implicated in cancers but is a major cause of genital warts [2, 20].

Additionally, the high-risk HPV39 which is detected in our study was not found in any of the 24 studies reviewed by Lawson et al. However, it was the major high-risk HPV detected in the recent UK study by Salman et al. [15, 21].

The p16 (cell cycle protein) expression is detected both in HPV-positive and negative cases in our cohorts. There is evidence that indicates strong expression as opposed to low or medium expression of p16 is associated with HPV biological activity and high expression of p16 can be used as a surrogate for an indication of transcriptional activity of HPV [7]. In our study, the tumor positive for the high-risk HPV16 was strongly positive for p16 and this may support previous observations made by other researchers that HPV16 may play an active role in the carcinogenesis of a small proportion of breast cancers [8, 15, 16].

## 5. Conclusion

The prevalence of HPV is low in Ethiopian breast cancer patients, but the role played by HPV in breast carcinogenesis among Ethiopian breast cancer patients cannot be commented based on these observations.

## Figures and Tables

**Table 1:** Baseline pathological characteristics of the study participants (*n* = 75).

Variables	*N* (%)
*Histological grade*	
I	5 (7)
II	22 (29)
III	25 (33)
Missing	23 (31)
*Histological type*	
Infiltrating ductal	45 (60)
Lobular	4 (5)
Others/not classified	17 (23)
Missing	9 (12)
*Stage*	
I	15 (20)
II	23 (31)
III	26 (35)
IV	1 (1)
Missing	10 (13)

**Table 2:** p16 protein expression among the study participants.

p16 expression	Number of cases	Percentage (%)
Neg	54	72
Pos (weak/moderate)	9	12
Pos (strong)	12	16
Total	75	100

## Data Availability

All materials used in the study are available and can be provided as necessary.
